# DNA Assembly Templated by Chiral Nanotube Lattices: From Helix to Rings

**DOI:** 10.1021/jacs.6c04280

**Published:** 2026-05-18

**Authors:** Ravi R. Sonani, Ali A. Alizadehmojarad, Nathaniel Hurley, Joshua Hihath, Michael S. Strano, Edward H. Egelman, Ming Zheng

**Affiliations:** † Department of Biochemistry and Molecular Genetics, 214841 University of Virginia, Charlottesville, Virginia 22908, United States; ‡ Department of Chemical Engineering, 2167 Massachusetts Institute of Technology, Cambridge, Massachusetts 02139, United States; § Materials Science and Engineering Division, 10833 National Institute of Standards and Technology, Gaithersburg, Maryland 20899, United States; ∥ Center for Bioelectronics and Biosensors, School of Electrical, Computer, and Energy Engineering, 7864 Arizona State University, Tempe, Arizona 85287, United States

## Abstract

Controlled assembly of DNA expands its utility in materials science. Most DNA-based architectures rely on Watson–Crick base pairing and stacking. Identifying additional programmable interactions could widen the design space. Single-stranded DNA (ssDNA) is known to adsorb on carbon nanotubes (CNTs) as sequence- and chirality-dependent helices, but direct structural evidence for a nanotube lattice-templating mechanism has been limited. Here we use cryo-electron microscopy to compare the assembly of the ssDNA sequence TTA TAT TAT ATT (ss65) on enantiomeric (6,5) CNTs. On the left-handed (+) (6,5) CNT, ss65 forms stacked, ring-like wraps with an axial repeat of 15.3 Å and with micrometer-scale coherence length. In contrast, on the right-handed (−) (6,5) CNT, ss65 adopts a conventional 1-start helical wrap with a helical pitch of ∼16.4 Å. These results indicate that the handedness of the underlying chiral lattice can bias ssDNA into distinct topologies (helix versus rings) and suggest a strategy for DNA assembly based on CNT lattice recognition, in addition to base pairing and stacking.

## Graphical abstract

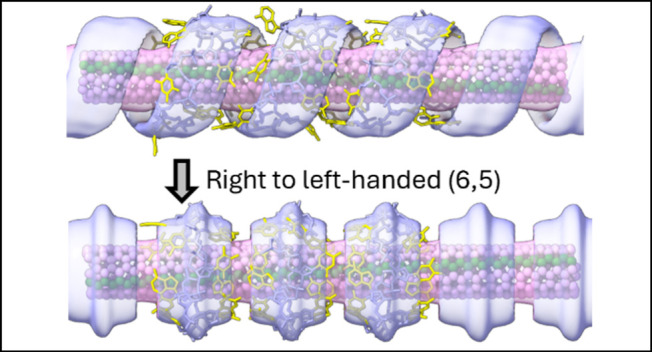

DNA assembly is increasingly used in materials science to create nanoscale structures with programmable geometry and function.^1^ Most current applications exploit naturally occurring DNA structural motifs stabilized by hydrogen bonding and base stacking as described in the Watson–Crick model.^2^ Expanding the repertoire of accessible motifs by harnessing DNA–inorganic interfacial interactions could enable new structural and functional possibilities. Progress in this field has been limited by the scarcity of experimentally resolved structures of DNA on inorganic surfaces. Carbon nanotubes (CNTs) provide an attractive platform for exploring such interactions because of their well-defined chiral lattices that can interact with single-stranded DNA (ssDNA). DNA-wrapped carbon nanotube hybrids (DNA-CNTs) have been used for CNT chirality sorting,^3–7^ controlled functionalization,^8,9^ and programmable assembly.^10,11^ Despite extensive use, however, the structural principles governing DNA organization on CNT surfaces remain incompletely understood. Here, we use cryo-electron microscopy (cryo-EM)^12–14^ to characterize the structures of DNA–CNT hybrids formed by a designed ssDNA sequence on enantiomeric (6,5) CNTs. By a judicious choice of the ssDNA sequence and enantiomeric (6,5) nanotubes, we isolate the role of the chiral CNT lattice in directing DNA assembly. We show that switching lattice handedness triggers a topological transition of ssDNA from continuous helical wrapping to a discrete, ring-stacked architecture with highly uniform axial periodicity. These results support the idea that lattice templating can complement base stacking and hydrogen bonding to create new DNA structural motifs and assemblies.

Our previous studies suggest that ssDNA wrapping depends strongly on the CNT lattice, enabling physical sorting by nanotube chirality,^5,6,15,16^ but quantitative structural evidence for this lattice effect has been limited. Using cryo-EM, we determine the wrapping structure of the ssDNA sequence TTA TAT TAT ATT (denoted ss65) on left- and right-handed (6,5) enantiomeric CNTs. The resulting hybrids, ss65­(+)­(6,5) and ss65(−)­(6,5), were purified as described in the Supporting Information. Optical absorbance and circular dichroism (CD) spectra of the two hybrids are shown in Figure 1 which corroborate well with previous results.^6,16^ We follow the convention^17^ to make CD-based CNT handedness assignment: (+) (6,5) is left-handed and (−) (6,5) is right-handed.

**Figure 1:**
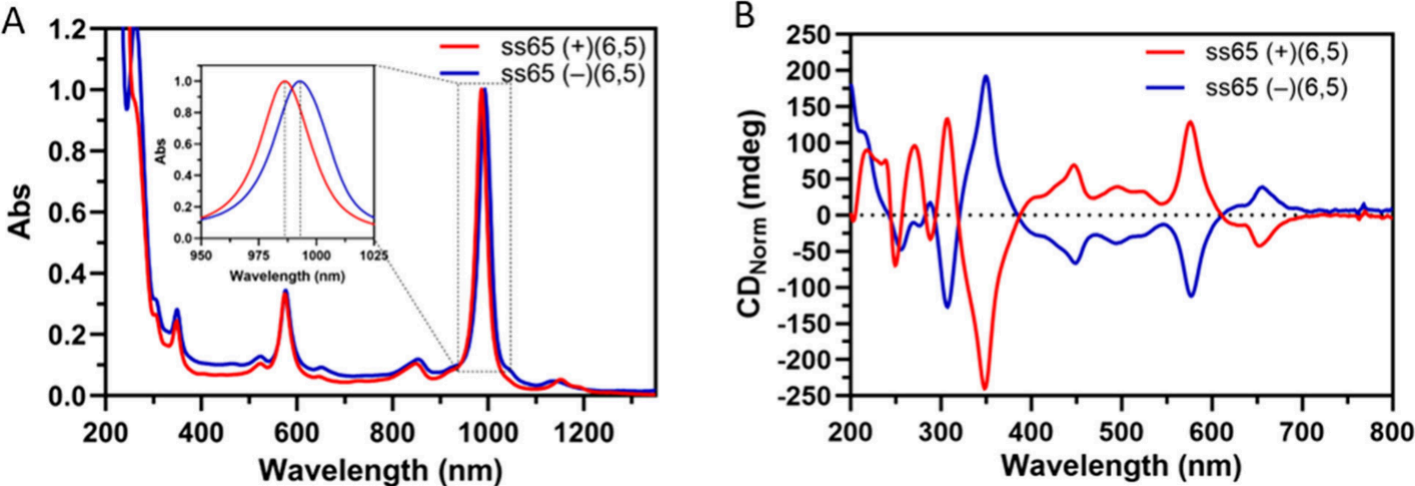
(A) UV–visible-NIR absorbance spectra (normalized at E11) of the ss65­(+)­(6,5) and ss65(−)­(6,5) samples used in the study. The inset highlights the peak shift between the two enantiomers. (B) Circular dichroism (CD) spectra of the ss65­(+)­(6,5) (E22 CDnorm = +128 mdeg) and ss65(−)­(6,5) (E22 CDnorm = – 111 mdeg).

Cryo-EM imaging revealed that both ss65­(+)­(6,5) and ss65(−)­(6,5) appear as straight, thin filaments dispersed across the vitreous ice (Figures 2 and 3). Each filament population displays uniform thickness and straightness over hundreds of nanometers. Because these specimens are thin and weakly scattering, the DNA wrapping pattern is not readily visible in raw micrographs (Figures 2A and 3A). To enhance the signal-to-noise ratio and extract consistent periodic information, we boxed thousands of short CNT segments and aligned-classified them to generate 2D class averages. The resulting 2D class averages - generated from 52127 and 21409 segments of ss65(−)­(6,5) and ss65­(+)­(6,5), respectively - revealed distinct axial periodicities for the two enantiomeric hybrids (Figures 2B and 3B). These averages represent a combined view of many individual projections, highlighting consistent structural features across many DNA-CNT assemblies.

**Figure 2:**
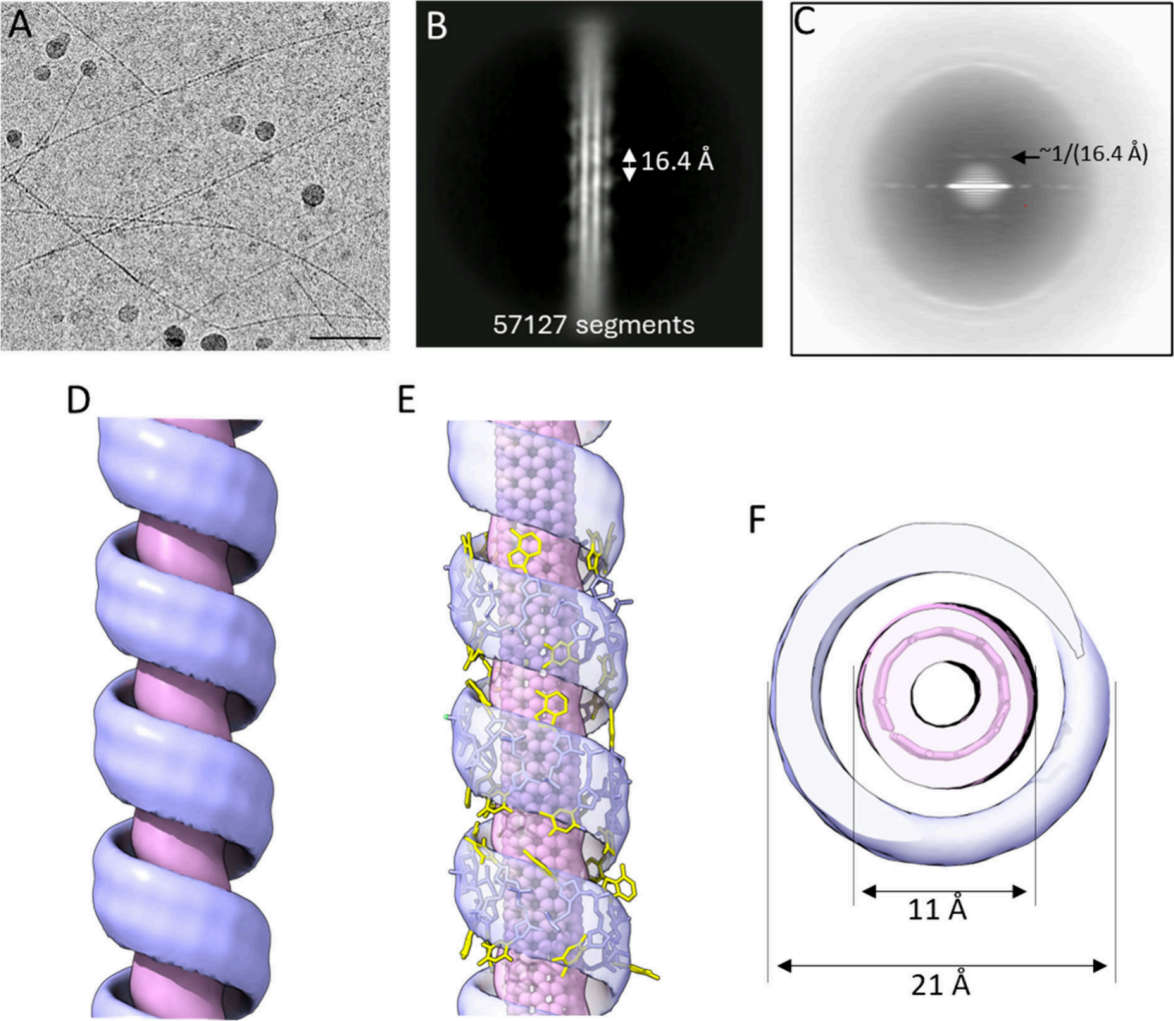
Cryo-EM of ss65(−)­(6,5). (A) Representative micrograph (scale bar = 50 nm), (B) 2D average, and (C) averaged power spectrum of vertically aligned segments. (D) 3D volume generated by helical averaging, (E) molecular dynamics flexible fitting (MDFF) derived ss65(−)­(6,5) model (DNA sugar–phosphate backbone in purple and nitrogenous bases in yellow) fitted into the cryo-EM map, and (F) top-view of cryo-EM map.

**Figure 3:**
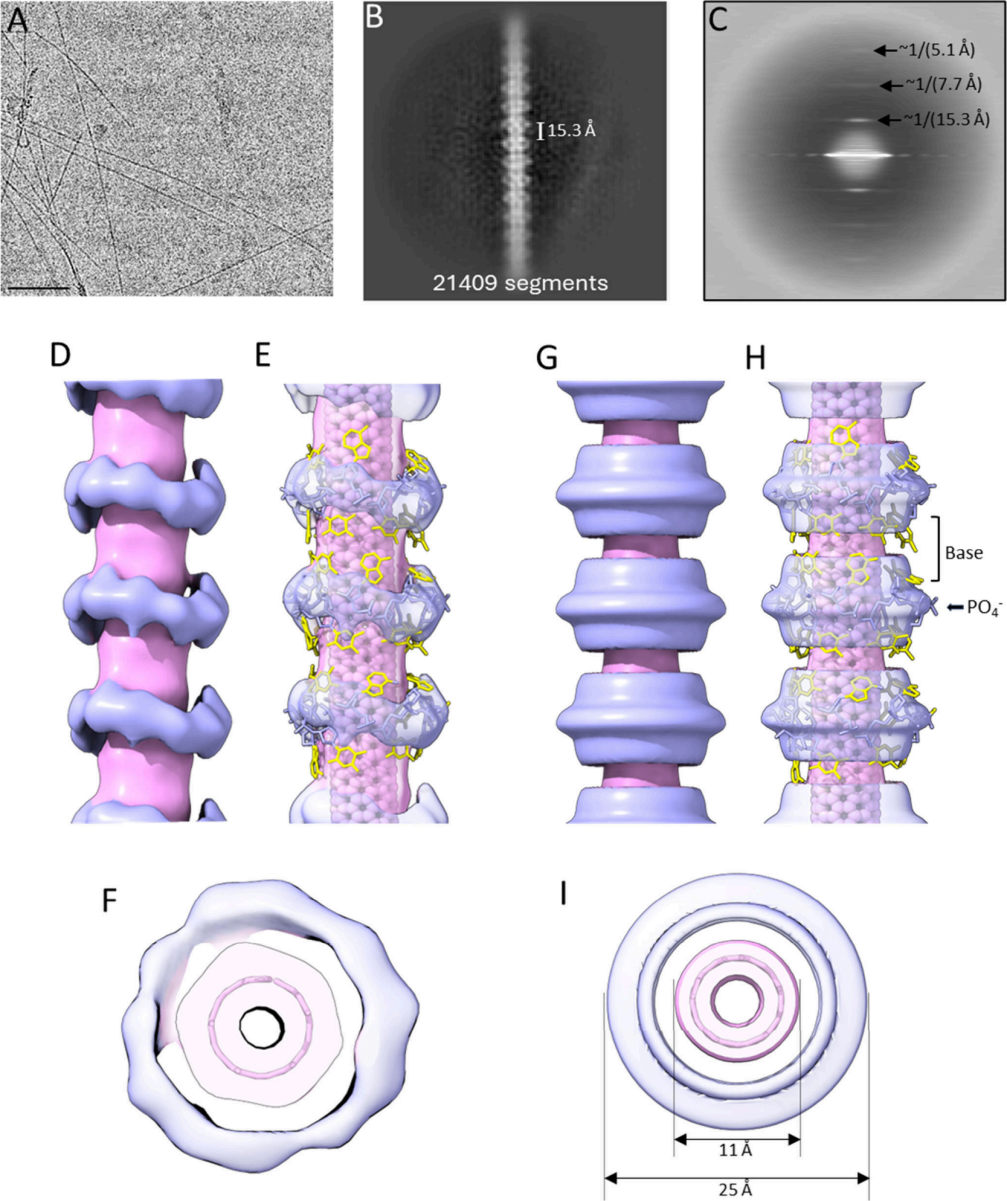
Cryo-EM of ss65­(+)­(6,5). (A) Representative micrograph (scale bar = 50 nm), (B) 2D average, and (C) averaged power spectrum of vertically aligned segments; (D-F) asymmetric reconstruction, (D) cryo-EM 3D volume, (E) MDFF derived ss65­(+)­(6,5) model (DNA phosphate-sugar backbone in purple and nitrogenous bases in yellow) fitted into the map, and (F) top-view of cryo-EM map. (G-I) Cylindrically averaged reconstruction, (G) 3D volume, (H) MDFF derived ss65­(+)­(6,5) model (DNA phosphate-sugar backbone in purple and nitrogenous bases in yellow) fitted into the map, and (I) top-view of cryo-EM map.

## Right-Handed (6, 5): Helical ssDNA Wrapping

The averaged power spectrum of vertically aligned ss65(−)­(6,5) segments (Figure 2C) shows a distinct layer line at ∼ 1/(16.4 Å) with a pair of symmetric off-meridional reflections. Given the (6,5) nanotube diameter, these features are consistent with a 1-start helical wrap of ssDNA around the nanotube having a pitch of ∼ 16.4 Å. The layer-line at 1/16.4 Å indicates that helical order is maintained over several turns at a resolution sufficient to resolve ssDNA strands. Initial asymmetric 3D reconstructions were noisy and lacked interpretable features, likely due to helical disorder at high resolution and the very weak scattering from these tubes. We therefore generated a helically averaged reconstruction using the 16.4 Å helical pitch derived from the power spectrum (Figures 2D and 2E). Although the map averages out high-resolution details such as individual base features, it clearly reveals density corresponding to the DNA wrapping around the nanotube. From the reconstructed volume, the diameter of the bare CNT was estimated to be ∼ 11 Å, while the ss65(−)­(6,5) hybrid measured ∼ 21 Å across (Figure 2F), consistent with a single ssDNA strand coating the nanotube. These findings are consistent with our prior work on the same DNA-CNT construct.^15^

## Left-Handed (6,5): Ring-Like ssDNA Wrapping

In contrast to the right-handed (6,5), the power spectrum of the ss65-wrapped left-handed (+) (6,5) (Figure 3C) shows a strong meridional reflection at 1/(15.3 Å), and lacks off-meridional reflections, indicating the absence of helical wrapping of DNA around the nanotube. Additional meridional peaks observed at ∼ 1/(7.7 Å) and 1/(5.1 Å) correspond to the second and third order reflections, respectively, of the 15.3 Å repeat, suggesting a highly regular axial stacking. An earlier atomic force microscopy (AFM) study of the same construct reported a similar periodicity (15.9 Å) but could not resolve the ring-like motif.^15^

An asymmetric reconstruction produced a 3D map revealing ring-shaped densities encircling the CNT (Figures 3D-3F), suggesting a mode of wrapping in which each DNA strand forms a complete loop around the nanotube. However, this reconstruction lacked sufficient resolution to resolve individual nucleotide features or detect any azimuthal twist between adjacent rings. To better visualize the average geometry, a cylindrically averaged reconstruction was generated by imposing a 15.3 Å axial rise and applying a high rotational symmetry (Figures 3G-3I). The resulting volume displays a distinct ridge of density at the midline of each ring, likely corresponding to the phosphate backbone, with flanking densities extending on both sides of this ridge that plausibly correspond to the nitrogenous bases (Figure 3H).

To estimate the coherence of the ring spacing, we simulated cumulative disorder of rings assuming the spacing of two adjacent rings is 15.3 Å ± σ, where σ is the deviation from the measured average spacing. We found that σ must be less than 0.2 Å to match the observed diffraction pattern in Figure 3C. If we define the coherence length (L) as the distance over which the accumulated positional uncertainty reaches half a period 7.65 Å, then $\sqrt{N}$ *0.2 Å = 7.65 Å, where N is the number of rings within L. This gives N ∼ 1,500, and L ∼ 2.3 μm.

Optical spectroscopy shows that the E11 peak position of the ss65­(+)­(6,5) is blue-shifted by 5 nm relative to that of the ss65(−)­(6,5) (Figure 1A), consistent with a change in the local dielectric environment^18^ determined by DNA wrapping structures. Additionally, our sorting experiments^6,16^ show that ss65­(+)­(6,5) is relatively more hydrophilic than ss65(−)­(6,5), which again suggests differences in DNA wrapping structure in the two (6,5) enantiomers. Together, these results from previous studies are consistent with a clear chirality-driven structural transition revealed here: right-handed (−) (6,5) promote continuous helical wrapping, whereas left-handed (+) (6,5) favor discrete ring stacking with highly uniform axial order.

## Molecular Dynamics Flexible Fitting (MDFF)

Both helical-^19^ and ring-like^20^ ssDNA conformations on CNTs have been suggested by molecular dynamics (MD) simulations, but direct comparison to experimental structures has been limited. In this work, our MD simulations of ss65 on the left- and right-handed (6,5) CNTs did not reproduce the experimentally observed axial periodicities for both cases (Supporting Information), suggesting that the current MD procedures and force field parameters may be insufficient for predicting the subtle free-energy differences governing ssDNA topologies on CNT lattices. We therefore used MDFF with the cryo-EM map as the constraints to derive plausible ss65 configurations for the right- and left-handed (6,5), as shown in Figure 2E and Figure 3E, respectively (also see Figures S6 and S7). This approach produced models consistent with the experimentally observed axial periodicities and folding topologies. Figures S6a and S6b show representative MDFF snapshots using a grid force-scaling factor (gscale) of 2 for the helical and ring DNA conformations around the (−) (6,5) and (+) (6,5), respectively. The resulting models reveal a more ordered and compact ring conformation on the (+) (6,5) compared to the helical conformation on the (−) (6,5).

## Hydrogen Bonding and Solvent Exposure

Using the MDFF-derived conformations as starting points, we performed short MD simulations with strong backbone restraints to compare hydrogen bonding and solvent organization. The ring conformation on (+) (6,5) forms more intra- and interstrand hydrogen bonds than the helical conformation on (−) (6,5) (Figure S6c) and exhibits a more compact arrangement. The resulting differences in solvent exposure and local dielectric environment provide a plausible microscopic basis for the observed E11 blue shift of ss65­(+)­(6,5) (Figure 1A).

## Lattice Templating Mechanism

The pronounced, handedness-dependent structural transition—together with the high regularity of the ring repeat—supports a role for chiral lattice recognition in guiding ssDNA organization on CNTs. Because base stacking and hydrogen bonding alone may be insufficient to enforce micrometer-scale axial registry, we postulate that the underlying CNT lattice provides an additional templating constraint. As shown in Figures 4A-4B, a chiral CNT lattice is characterized by three symmetry-related zigzag or armchair helices. We propose a templating mechanism in which a given reference point on each ssDNA strand (e.g., the 5′ end) registers with one of the helical lines on the CNT surface, and adjacent strands adopt axial positions that preserve this registration (Figure 4C). For ss65­(+)­(6,5), we suggest that DNA rings are in registration with the Z3 helix that has a helical pitch (15.3 Å) the same as the 15.3 Å ring–ring separation. As a result of this “accidental” length scale overlap, adjacent rings would have very small azimuthal rotations relative to each other and the hypothetical helical pattern formed by the ring array has a pitch too large to be observable using short segments. Direct visualization of atomic-scale registration will require improved sample homogeneity and averaging of much longer segments, which we expect to be achievable as DNA-CNT purification and image analysis methods continue to advance.

**Figure 4:**
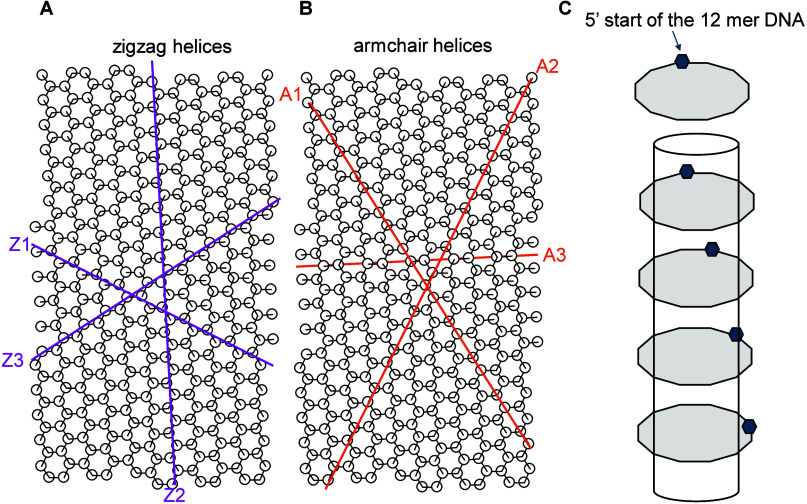
Proposed lattice-templating model for handedness-dependent ssDNA organization on (6,5) CNTs. (A–B) Symmetry-related helical lines on (6,5) lattices. (C) Schematic illustrating axial registration of equivalent DNA reference points with a lattice helix.

In summary, our cryo-EM structures reveal a handedness-controlled topological switch for a single ssDNA sequence on enantiomeric (6,5) nanotubes - helical wrapping on (−) (6,5) versus ring stacking on (+) (6,5), and suggest chiral lattice templating as a programmable interaction for DNA assembly beyond base pairing and stacking.

## Supplementary Material



## Notes

Detailed experimental methods, purification procedures, additional cryo-EM class averages and reconstructions, and MD/MDFF analyses (PDF)

## Abbreviations

CNT: Carbon nanotubes
UV–vis NIR: Ultraviolet visible near-infrared
Cryo-EM: cryogenic electron microscopy
