# Supplementary material for: DNA Assembly Templated by Chiral Nanotube Lattices: From Helix to Rings
Source: J Am Chem Soc. 2026 May 18;148(21):21217–22. doi: 10.1021/jacs.6c04280 (PMC13244465; doi:10.1021/jacs.6c04280)
Supplement: Supplementary file 1 [file ja6c04280_si_001.pdf]

Supplemental Materials

DNA Assembly Templated by Chiral Nanotube Lattices: From Helix to Rings

Ravi Sonani[1#], Ali A Alizadehmojarad[2#], Nathaniel Hurley[3#], Joshua Hihath[4], Michael S Strano[2*], Edward H Egelman[1*], Ming Zheng[3*]

[1]Department of Biochemistry and Molecular Genetics, University of Virginia, Charlottesville, VA 22908, United States
[2]Department of Chemical Engineering, Massachusetts Institute of Technology, Cambridge, MA 02139, United States
[3]Materials Science and Engineering Division, National Institute of Standards and Technology, Gaithersburg, MD 20899, United States
[4]Center for Bioelectronics and Biosensors, School of Electrical, Computer, and Energy Engineering, Arizona State University, Tempe, AZ 85287, United States

[#]Co-first authors
*Co-corresponding authors: ming.zheng@nist.gov (ORCID ID: 0000-0002-8058-1348); egelman@virginia.edu; strano@mit.edu (ORCID ID: 0000-0003-2944-808X)

This supplementary material contains:

- Materials and Methods including Table S1
- Supplementary Results & Discussion including Table S2, and Figures (S1-S8)

Certain equipment, instruments or materials are identified in this paper in order to adequately specify the experimental details. Such identification does not imply recommendation by the National Institute of Standards and Technology (NIST) nor does it imply the materials are necessarily the best available for the purpose. Unless noted otherwise, all reagents were obtained from standard sources.

Materials and Methods

1. Sample preparation and characterization

1.1. Materials

CoMoCAT SWCNT powder SG65i grade, lot no. SG65i-L46 were acquired from Southwest Nanotechnologies. Chemicals including sodium thiocyanate (NaSCN, 98.0%), polyethylene glycol (6000 MW), sodium phosphate dibasic heptahydrate (98.0-102.0%), sodium phosphate monobasic monohydrate (99.5%), potassium phosphate monobasic, potassium phosphate dibasic, sodium carbonate (99.0%), potassium carbonate (99.0%), were purchased from Sigma-Aldrich. Oligonucleotide deoxyribose nucleic acid (DNA) was obtained from IDT. All chemicals were used without further purification.

1.2. Procedures

1.2.1. DNA-SWCNT Dispersion

DNA-SWCNT dispersions were prepared following a previously published method with some modifications.[1] In short, 1 mg SG65i soot and 2.5 mg DNA (ss65: TTATATTATATT) were added to a 1 mL aqueous solution of 30 mM NaCl. The mixture in a 2 mL Eppendorf tube was placed in an ice-water bath and then ultrasonicated (model VCX 130, Sonics and Materials, Inc.) for 90 mins. The resulting dispersion was then centrifugated for 1 minute at 17,000 ×g and the supernatant was transferred to a new vial, the pellet was discarded.

1.2.2. ss65(–)(6,5) Purification

The extraction of ss65(–)(6,5) was performed using polyethylene glycol (PEG) 6K / 3:1 sodium-potassium phosphate buffer (NaKPB) aqueous two-phase (ATP) system. See **Table S1** for the ATP recipe. The 3:1 molar ratio of Na:KPB stock solution was made by a mixing 3:1 volume ratio of 3M pH 7 NaPB (9.3 g $Na_2HPO_4*_7H_2O$ and 3.51 g $NaH_2PO_4*H_2O$ in 20 mL water) and 3M pH 7 KPB (5.61 g K_2HPO_4 and 3.78 g KH_2PO_4 in 20 mL water). In a typical separation, 7 volumes (350 µL) of ATP are mixed with 3 volumes (150 µL) of DNA-SWCNTs. The ss65-(–)(6,5) is extracted from the PEG rich top phase after vortexing and centrifugation. The extracted top phase was centrifuged for 10 min at 17,000 ×g. To the extracted supernatant 0.7 M NaSCN (final concentration) was added to precipitate out the ss65-(–) (6,5). The pellet was collected after 10 min centrifugation and resuspended in a 20 mM pH 7 NaPB buffer with 0.1 mg/mL ss65 DNA.

1.2.3. ss65(+)(6,5) Purification

The extraction of ss65(+)(6,5) from the bottom phase requires different steps than the ss65-(–)(6,5). First 7:3 and 10:0 ATP systems (**Table S1**) made using sodium carbonate and potassium carbonate were prepared. The process to make these followed the previously reported method.[2] In short, stock solutions of 40 wt% 6K PEG and 1:5 Na:K-carbonate (made with 2.5 mL of 50

wt% K_2CO_3 (7.235 M) and 1.15 mL 25wt% Na_2CO_3 (3.145 M)) are prepared. The stock solutions are then mixed using the volumes listed in **Table S1**.

To separate ss65(+)(6,5), 350 µL of 7:3 ATP and 150 µL DNA-SWCNT dispersion are mixed and vortexed for 10 seconds followed by 1 min centrifugation at 17,000 ×g. The volume of the separation can be scaled up or down as long as the 7:3 ratio is maintained. It was observed that carbonate salts can result in rapid aggregation of DNA-SWCNTs. To minimize aggregation all separation steps should be done as quickly as possible. After centrifugation, ss65(+)(6,5) is partitioned in the bottom phase. The top phase and any material trapped at the interface is extracted and discarded. The bottom phase is diluted with 1:1 volume of pH 6.5 PEG/ NaPB 10:0 bottom phase and 1 uL 10 mg/mL DNA. This addition reduces the pH and increases the amount of free DNA in solution. Sodium thiocyanate (NaSCN) is then added to a final concentration of 2.5 M and vortexed to ensure an even mixture. Centrifuge the mixture for 15 min at 17,000 ×g, rotate vial 180 degrees, and centrifuge 15 min more. A pellet containing the ss65(+)(6,5) should form at the bottom of the vial. The pellet is then redispersed in 20 mM pH 7 NaPB with 0.1 mg/mL DNA by vortexing and bath sonication. Finally, the sample was centrifuged 10 mins at 17,000 ×g to remove remaining bundles. The supernatant containing ss65(+)(6,5) was extracted and transferred to a clean vial.

Table S1. ATP system recipes

Molar Salt Ratio	pH	PEG 6k, 40 wt% (mL)	Salt volume (mL)	Water (mL)
3:1 Na:K- phosphate	8.0	1.20	1.30<br>(2M NaPB/ 3M K PB pH 8)	**7:3**- 1.07<br>**10:0**- 2.60
1:0 Na:K- phosphate	6.5	2.39	2.50<br>Na: 2M	**14:5**- 2.57<br>**10:0**- 5.23
1:5 Na:K-carbonate	11-13	2.2	1.3<br>(1:5 Na:K-carbonate)	**7:3**- 2.31<br>**10:0**- 4.80

1.3. Instrumentation

1.3.1. Ultraviolet-visible Near infrared (UV-Vis-NIR) spectroscopy

UV-Vis-NIR absorbance measurements were carried out on a Cary 5000 spectrophotometer. Absorbance measurements were taken from 200-1350 nm with a data interval of 1 nm and scan speed of 600 nm/min.

1.3.2. Circular dichroism (CD)

CD measurements were taken on a Jasco J-1500 CD Spectrophotometer with the following set of parameters: bandwidth: 2 nm; wavelength range: 800-200 nm; data pitch:1 nm; and 4 accumulations at a scan speed of 100 nm/min. Measurements were taken using a 2 mm pathlength quartz cuvette.

1.3.3. Fluorescence

2D Fluorescence maps were taken on a Model NS Super Chiroptical NanoSpectralyzer, with an excitation range of 405-850 nm, step size of 5 nm, integration time of 2000 ms, and an average of 6 scans. Dispersions of about 0.15 OD @ E11 ss65-(6,5) samples were prepared and 120 uL were aliquoted into a 1 cm pathlength cuvette. To measure DOC wrapped samples a final concentration of 1% DOC was added to the DNA wrapped (6,5) followed by adding a final concentration of 10 mM NaOH.

2. Cryo-Electron Microscopy

2.1 Vitrification of CNT-DNA samples on lacey carbon grid

Vitrification of the sample was done using an EM GP Plunge Freezer (Leica). The 3 μL of the CNT–DNA suspension was applied to a glow-discharged lacey carbon grid. After application, excess sample was blotted from the back side of the grid for 3.5 seconds using Whatman Grade 1 filter paper, to create a thin, uniform layer of sample. The grids were rapidly plunge-frozen in liquid ethane and subsequently stored in liquid nitrogen until cryo-EM imaging.

2.2 Data Collection and Image Preprocessing

Vitrified grids were first screened on a 200 keV Glacios transmission electron microscope (Thermo Fisher Scientific) to identify areas with optimal ice thickness and well-dispersed CNTs. Grids meeting these criteria were subsequently transferred to a 300 keV Titan Krios microscope (Thermo Fisher Scientific) equipped with a K3 direct electron detector (Gatan, Inc.) for high-resolution data acquisition. Dose-fractionated movies were recorded with a total electron exposure of ~50 $e^-/Å^2$ and a calibrated pixel size of 0.652 Å. Raw movie frames were corrected for both beam-induced and stage motions using the *patch motion correction* routine in cryoSPARC.[3] The resulting motion-corrected micrographs were then subjected to contrast transfer function (CTF) estimation via the *patch CTF estimation* module within the same software.

2.3 Data processing

Data processing was carried out using cryoSPARC. Initial CNT segments were manually boxed and extracted from a representative subset of micrographs to generate 2D class averages, which were subsequently used as templates for automated particle selection via the *Filament Tracer* tool. Two-dimensional classification was performed to eliminate poor-quality picks, classify particles, and obtain vertically aligned averages for each class. Power spectra from the best-resolved classes were computed using the *Generate Power Spectra* job within cryoSPARC and further analyzed in EMAN2.[4] The 52127 and 21409 segments of ss65(-)(6,5) and ss65(+)(6,5) were used for power spectra generation, respectively. Asymmetric and helically averaged map of ss65(+)(6,5), and cylindrically averaged map of ss65(-)(6,5) were produced using *Ab-initio Reconstruction*, *Homogeneous Refinement*, and *Helical Refinement* modules in cryoSPARC, as needed.

3. Molecular dynamics (MD) simulation

MD simulations of the TTATATTATATT DNA oligonucleotide wrapping either the (-)(6,5) or (+)(6,5) SWCNT enantiomer were performed to probe its conformational behavior on these nanotubes. All systems were prepared and visualized using VMD and its associated plugins, including Solvate and Ionize, for solvating and neutralizing the DNA-SWCNT complexes.[5] The TIP3P water model was used to represent solvent molecules, and the NaCl concentration was set to 30 mM to match the experimental conditions. All simulations were carried out using NAMD 2.14 with the CHARMM36 force field.[6–8] Production runs were performed for at least 200 ns with a 2-fs integration timestep in the NPT ensemble, maintaining a temperature of 300 K and a pressure of 1 bar. Periodic boundary conditions were applied in all directions, and long-range electrostatics were computed using the particle mesh Ewald (PME) method.[9] Each system underwent 1000 steps of energy minimization to remove steric clashes and unfavorable contacts prior to equilibration.

In simulations involving a single DNA strand, both left-handed (LH) and right-handed (RH) DNA conformations were used to wrap the (6,5) SWCNT enantiomers to examine the dependence of DNA structure on the initial configuration. The end-to-end distance of each DNA conformation was computed for every frame as the difference in the z-coordinates between the centers of mass (COMs) of the terminal residues of the strand. In simulations involving three DNA strands, pitch distances were calculated as the z-coordinate difference between the COMs of two adjacent strands. These values were computed using four different atom selections: all DNA atoms, backbone atoms only, base atoms only, and phosphate-backbone atoms only.

4. Replica exchange MD (REMD) simulation

REMD simulations have previously been used to extensively explore DNA conformations around SWCNTs.[10–13] Therefore, we performed a 100-ns REMD simulation with a 2-fs timestep to investigate the probability of observing a helical structure around the (-)(6,5) SWCNT since this structure was suggested in our standard MD simulations for (-)(6,5) SWCNT versus unobserved ring structure in standard MD simulations for (+)(6,5) SWCNT. For this purpose, the final, well-equilibrated MD snapshot of the LH-DNA wrapping the (-)(6,5) SWCNT was used as the initial structure, while the COM of the DNA was constrained with a harmonic force constant of 1 kcal/(mol·Å^2). Prior to initiating the REMD run, the system was energy-minimized and then equilibrated for 100 ps in the NVT ensemble to bring it to room temperature. The REMD system contained 16,000 atoms and was simulated in the NVT ensemble using 80 replicas spanning a temperature range of 290-727 K. An average exchange acceptance ratio of 25% was achieved with an exchange attempt interval of 2 ps. Snapshots were saved every 2 ps, yielding 50,000 configurations per replica; only the final 25,000 snapshots from each replica were used for analyzing DNA conformations.

5. Molecular dynamics flexible fitting (MDFF) simulation

MDFF simulations were prepared and performed using the NAMD 2.14 package and VMD, in explicit solvent, while maintaining the temperature at 300 K via a Langevin thermostat.[14] To generate the MDFF potential map, the cryo-EM electron density maps were first converted into a VMD-readable format. Each map was truncated to include only the nanotube and DNA density, and a 60 Å segment along the *z*-direction was extracted for use as the cryo-EM reference in the MDFF simulations for both the helical and ring DNA systems. A 3D potential map for MDFF was then constructed from each truncated density map. The initial DNA-SWCNT structures were rigid-body docked into the corresponding density maps, after which atomic masses were assigned as weighting factors for the external potential energy fields. The grid force-scaling factor, gscale, which determines the strength of coupling between the atoms and the cryo-EM-derived potential, was varied to identify optimal fitting conditions. MDFF simulations were run for 10 ns with a 1-fs timestep, testing gscale values of 0.3, 2, 5, and 10. No restraining forces were applied to the structures during these simulations. Among the tested values, gscale = 2 produced the best fit to the cryo-EM density. To further probe hydrogen bond formations and water organization around the (6,5) SWCNT enantiomers wrapped by helical or ring DNA, short 2-ns standard MD simulations were performed using the final MDFF snapshot as the initial structure, with the DNA backbone strongly constrained.

6. Modeling power spectra

Density maps for the MDFF-derived models-comprising only backbone atoms, only base atoms, or only phosphate-backbone atoms of DNA wrapped around (–)(6,5) and (+)(6,5) CNTs-were generated using the *molmap* function in ChimeraX [15] with a 3 Å filter. The resulting maps were resampled to match the sampling of the experimental cryo-EM maps. Power spectra from the resampled maps were then calculated using SPIDER.[16]

Supplementary Results & Discussion

MD simulation results

Isolating highly pure (-)(6,5) and (+)(6,5) SWCNT enantiomers using the TTATATTATATT DNA oligonucleotide enabled the capture of two distinct DNA conformations, helical and ring DNAs around each (6,5) nanotube, as revealed by cryo-EM imaging. This represents the first experimental observation of a ring DNA conformation on SWCNTs, a structure that had previously been predicted to form around the larger-diameter (9,4) SWCNT (~50% larger diameter than (6,5)), using standard MD simulations and enhanced sampling methods.[10,12] Our current experimental observations indicate that ring formation can also occur on small-diameter nanotubes such as the (6,5) chirality. However, resolving the complete DNA structure, including individual bases, was not possible in cryo-EM images, which can be likely due to incredibly thin nature of the DNA-CNT and finer heterogeneity in the samples. Therefore, multiple classical molecular dynamics (MD) simulation approaches were used to investigate DNA conformations around LH and RH (6,5) nanotube enantiomers in greater detail. Standard MD simulations at room temperature were performed to examine how SWCNT handedness affects DNA wrapping. These fixed-temperature simulations showed that the final conformation of the TTATATTATATT oligo depends strongly on its initial configuration. As opposed to cryo-EM results, no ring formation was observed for the (+)(6,5) SWCNT regardless of whether the initial DNA structure was right-handed or left-handed. **Figure S1** presents representative MD snapshots and end-to-end distance distributions, confirming the absence of ring conformations around the (+)(6,5) enantiomer. Because fixed-temperature MD simulation is susceptible to kinetic trapping, which limits exploration of the full conformational landscape, we performed replica-exchange MD (REMD) simulations for helical DNA around (-)(6,5) enantiomer over a temperature range of 273-720 K. The end-to-end distance distribution at 300 K extracted from REMD simulation (**Figure S2**) demonstrates that DNA conformations become fully decorrelated from their initial structures and dynamically interchange among four predominant states: left-handed helix, right-handed helix, compact helix, and ring. REMD simulations suggest that helical DNA structures are observable, consistent with experimental results, while ring conformations also appear as transient states between right-handed and left-handed DNA helices. In contrast to the helical DNA observed around (−)(6,5), it is expected that REMD simulations for (+)(6,5) may not capture ring conformations, as they did not appear in long standard MD simulations performed for (+)(6,5), despite the enhanced sampling capability of REMD.

To better approximate the experimental observations, 6nm segments of the (-)(6,5) and (+)(6,5) SWCNT enantiomers were initially wrapped with three helical and three ring DNA structures, respectively. The final MD snapshots of these systems are shown in **Figure S3**. The simulations indicate that helical DNA conformations remain stable over 200 ns, with the bases remaining relaxed and adsorbed on the nanotube surface. In contrast, the ring DNA conformations around the (+)(6,5) SWCNT were significantly perturbed, leading to disordered structures and partial desorption of several DNA bases. These disordered ring conformations are inconsistent with the cryo-EM observations and may be due to the fact that the ring conformation may not be energetically favorable under the current, unmodified force-field parameters. Pitch distances were also computed during the simulations, defined as the distance between the centers

of mass (COMs) of two adjacent DNA strands. Because each system contains three DNA strands, two pitch distance values are obtained at each simulation timestep. As shown in **Figure S3**, the pitch distances of the helical and ring DNA strands fluctuate around 18.7 Å. These values are approximately 2-3 Å larger than the experimental pitch distances of 16.4 Å (helical) and 15.3 Å (ring). Additionally, several alternative initial configurations for the ring structures such as rotating individual DNA strands to bring them closer together were tested in separate MD simulations. In all cases, the DNA strands became disordered and deviated from the expected ring conformation.

Resolving atomistic DNA conformations on (-)(6,5) and (+)(6,5) SWCNTs using molecular dynamics flexible fitting (MDFF)

Standard MD simulations reproduced DNA helical conformation revealed by cryo-EM measurements for the (-)(6,5) SWCNT, even without customized force-field parameters. The pitch distance was off by ~2 Å, however. In sharp contrast, neither the ring DNA conformation around the (+)(6,5) SWCNT nor its corresponding pitch distances were captured using standard MD simulations. To address these limitations, we employed molecular dynamics flexible fitting (MDFF) to fit the DNA-SWCNT structures into the cryo-EM electron density maps, treating the experimental maps as ground-truth references.

Figures S4 and **S5** show representative MDFF snapshots using a gscale of 2 for the helical and ring DNA conformations around the (-)(6,5) and (+)(6,5) SWCNTs, respectively. MDFF successfully fits the DNA structures into the experimental cryo-EM densities. The resulting models reveal a more ordered and compact ring conformation on the (+)(6,5) SWCNT compared to the helical conformation on the (-)(6,5) SWCNT. The pitch distances calculated from the COM of the two DNA strands were 17.97 Å for the helical structure and 15.93 Å for the ring structure. These values differ from the experimental values by approximately 0.4-1.6 Å. Because the specific atomic features contributing to pitch measurements in the cryo-EM experiment are not explicitly known, we computed pitch distances using four definitions based on all DNA atoms, only backbone atoms, only bases' atoms, and only phosphate backbone atoms and obtained no significant differences. This confirms that the discrepancies between computational and experimental pitch distances do not arise from the choice of pitch-distance definition in the MDFF analysis.

We further modeled power spectra using MDFF-derived model structures with gscale values of 0.5, 2, and 5. Four structural representations were analyzed: all DNA atoms, backbone atoms only, bases only, and phosphate groups' atoms only. The MDFF-derived structures and their corresponding simulated power spectra for the helical and ring conformations are shown in **Figures S4** and **S5**. These results indicate that simulated power spectra calculated using models containing only DNA backbone atoms (from MDFF with gscale = 2) yielded pitch distances of 16.5 Å (helical) and 16.1 Å (ring). Consequently, the discrepancies relative to experiment were reduced to 0.1-0.6 Å, compared with the 0.4-1.6 Å differences obtained directly from MDFF structural analysis. Overall, these findings suggest that pitch distances should be computed from simulated power spectra derived from MDFF backbone-only models, as this approach yields the closest agreement between simulations and experiments.

Hydrogen bonding and surface coverage of DNA on SWCNTs depend on DNA conformation

The compactness of ring versus helical DNA strands on (6,5) SWCNT enantiomers directly influences both the number of inter- and intrastrand hydrogen bonds and the degree of nanotube surface coverage. As described above, cryo-EM power spectra shows that ring DNA structures on the (+)(6,5) SWCNT has a smaller pitch distances than helical DNA structures, indicating that ring DNAs adopt more compact conformations. One consequence of this increased compactness is the formation of a greater total number of hydrogen bonds in ring DNAs compared to helical DNAs, as visually evident in **Figures S6a** and **S6b**. The MDFF snapshots obtained with a gscale of 2 were then used as initial structures for short 2-ns MD simulations in which the DNA backbones were strongly constrained. The distribution curves of the total number of hydrogen bonds for both conformations are shown in **Figure S6c**. These results confirm that ring DNAs on the (+)(6,5) SWCNT form a larger number of hydrogen bonds than helical DNAs on the (-)(6,5) SWCNT.

The compactness of the DNA structures also alters the organization of water molecules around the nanotube. **Figure S7** illustrates these effects visually and quantitatively for both DNA conformations on the (6,5) SWCNT enantiomers. **Figure S7a** shows representative MD simulation snapshots from the 2-ns MD simulation as described above. To visualize water residence stability, water molecules within the first two hydration layers around the SWCNT are displayed for three snapshots over a 1 ns simulation time. These snapshots reveal that water molecules occupy the gaps between DNA strands. Notably, the gaps in the compact ring structure on the (+)(6,5) SWCNT are smaller than those in the helical structure on the (-)(6,5) SWCNT, consistent with the smaller pitch distances measured for ring DNAs. This trend is quantified in **Figure S7b**, which shows normalized 2D water-population maps for the first two hydration layers, demonstrating that the water density around the (-)(6,5) SWCNT wrapped with helical DNA is greater than around the (+)(6,5) SWCNT wrapped with ring DNA.

Because these simulations closely resemble the experimental conditions, the results provide valuable insight into the solvent exposure of (6,5) SWCNT enantiomers and offer a mechanistic explanation for their solvatochromic behavior. Our MD simulations incorporating realistic DNA conformations suggest that the compact ring DNA on the (+)(6,5) SWCNT leads to reduced solvent exposure relative to the helical DNA on the (-)(6,5) SWCNT. This reduced exposure is consistent with the experimentally observed blue-shifted emission wavelength of the (+)(6,5) enantiomer. Importantly, these findings represent the first direct computational evidence linking DNA conformational differences to solvatochromic shifts in SWCNT enantiomers.

Occupancy of ring and helical DNAs on (6,5) enantiomers

Not only did our cryo-EM results reveal structural differences of ss(6,5) DNA on the two (6,5) enantiomers, but they also enabled us to perform MDFF simulations and directly quantify the number of DNA strands wrapping around a single nanotube, which has been a long-standing challenge. Our MDFF simulations suggest that ss(6,5) DNA covers 1.7 nm (corresponding to 162 carbon atoms) and 2.2 nm (corresponding to 197 carbon atoms) of (+)(6,5) and (−)(6,5)

SWCNTs, respectively. These values correspond to DNA/SWCNT mass ratios of 1.8 and 1.5 for (−)(6,5) and (+)(6,5), respectively. Table S2 summarizes the details of the DNA/SWCNT mass ratio calculations for the (6,5) enantiomers. These evaluated DNA/SWCNT mass ratios are in close agreement with previously reported bulk and solution measurements,[13] which establish a DNA/SWCNT mass ratio range of 1.2–1.8.

Table S2. SWCNT length covered by ring and helical ss(6,5) DNA and their corresponding DNA/SWCNT Mass Ratios

	**SWCNT length covered by DNA (nm)**	**#SWCNT atoms covered by DNA**	**Mass (g/mole)**	**DNA/SWCNT mass ratio**	**Average DNA/SWCNT mass ratio (Ref. 13)***
**ss65 DNA**	-	-	3624.5	-	-
**(+)(6,5) wrapped by ring**	1.7	162	1944	1.8	~ 1.2
**(-)(6,5) wrapped by helix**	2.2	197	2364	1.5	

* This average DNA/SWCNT mass ratio was calculated by averaging six DNA/SWCNT mass ratios determined for three different SWCNT batches, as reported in Table 1 of Ref. 13.

Supplementary Figures

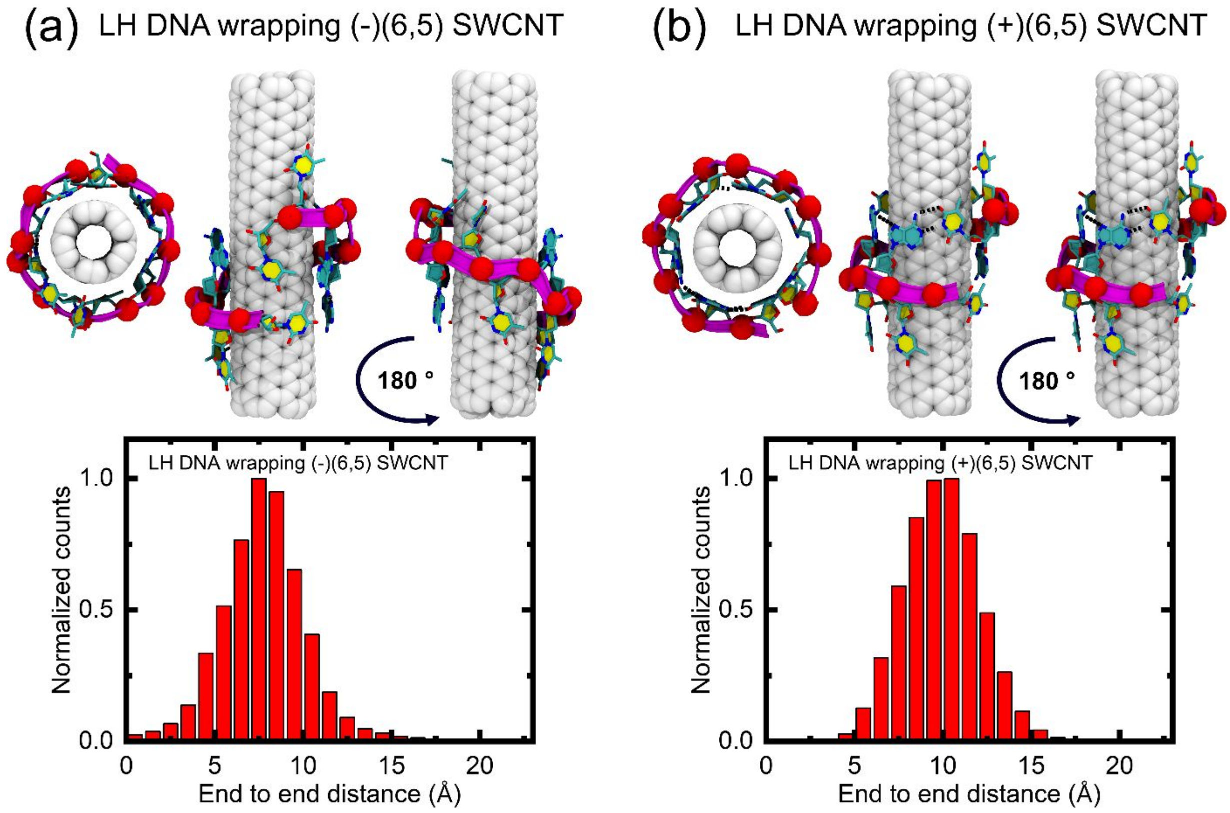

Figure S1. Standard MD simulations of a single DNA strand wrapping around (6,5) SWCNT enantiomers. Top panels show front and side views of the final-frame snapshots for (a) a single left-handed (LH) DNA wrapping the right-handed (-)(6,5) SWCNT, (b) a single left-handed (LH) DNA wrapping the left-handed (+)(6,5) SWCNT. Bottom panels show the corresponding normalized distributions of the end-to-end distances of the DNA strands over the last 50 ns of each simulation. SWCNT carbon atoms and the oxygen, nitrogen, and carbon atoms of the DNA are shown in white, red, blue, and cyan, respectively. Phosphorus atoms are displayed using red van der Waals spheres. Water molecules are omitted for clarity.

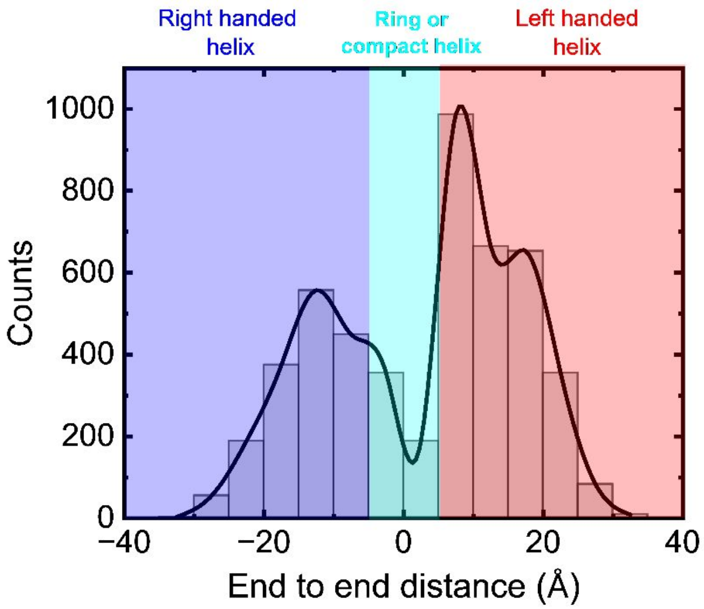

Figure S2. End-to-end distance distribution of the DNA strand around the (-)(6,5) SWCNT, computed from the last 50 ns of the replica-exchange MD (REMD) simulation at room temperature. The DNA structure dynamically alternates between right-handed (blue) and left-handed (red) helical conformations on the (-)(6,5) SWCNT. Transient conformations, such as ring-like and compact-helix structures, are also sampled during these transitions.

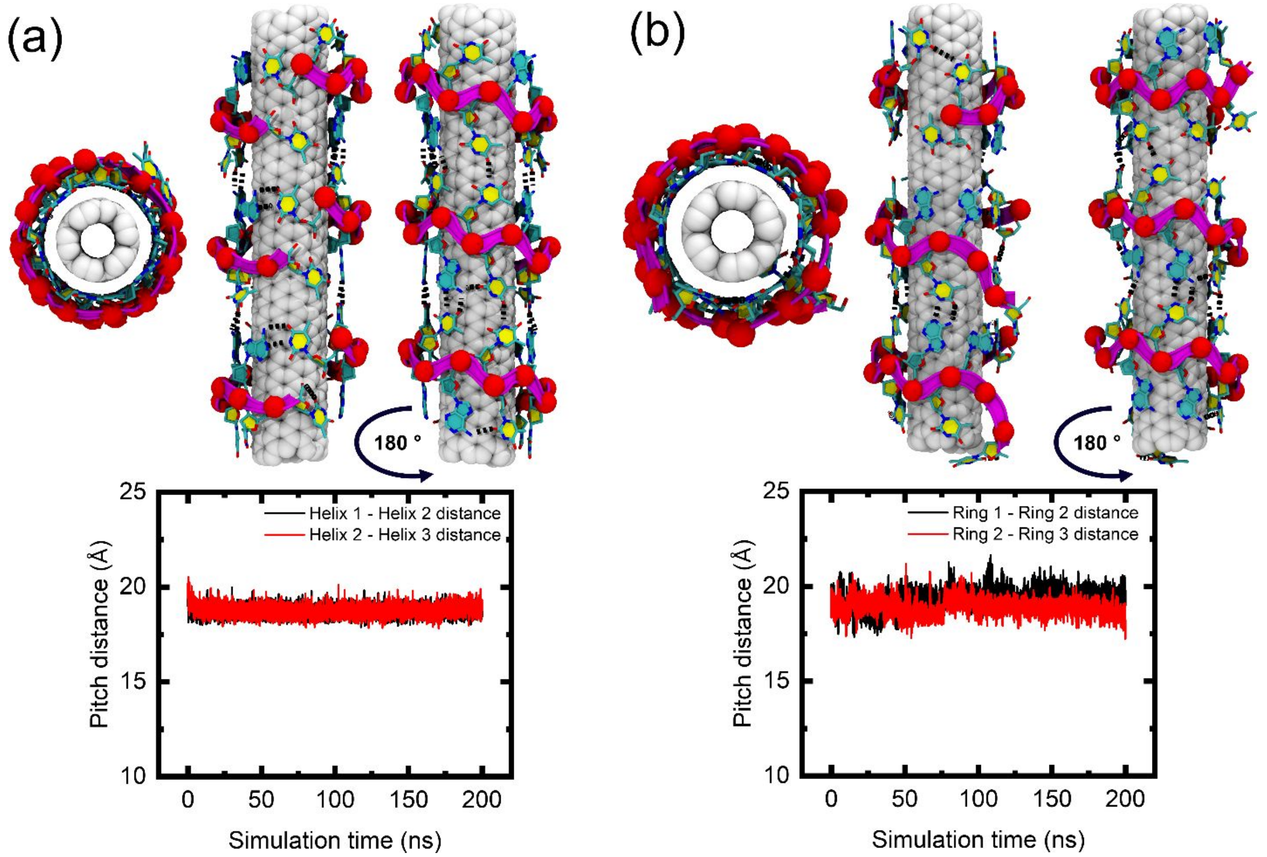

Figure S3. Pitch distances of helical and ring DNA strands wrapping the (-)(6,5) and (+)(6,5) SWCNTs. Top panels show equilibrated MD snapshots of (a) the (-)(6,5) SWCNT and (b) the (+)(6,5) SWCNT wrapped by three helical and three ring DNA strands, respectively. Bottom panels show the corresponding pitch distances over 200 ns of simulation. Pitch distance is defined as the z-direction distance between the centers of mass (COMs) of two adjacent DNA oligos. Numbering of helical and ring DNA strands begins with the bottom strand (Helix/Ring 1). The color scheme matches that in Figure S1. Hydrogen bonds are indicated with black dashed lines.

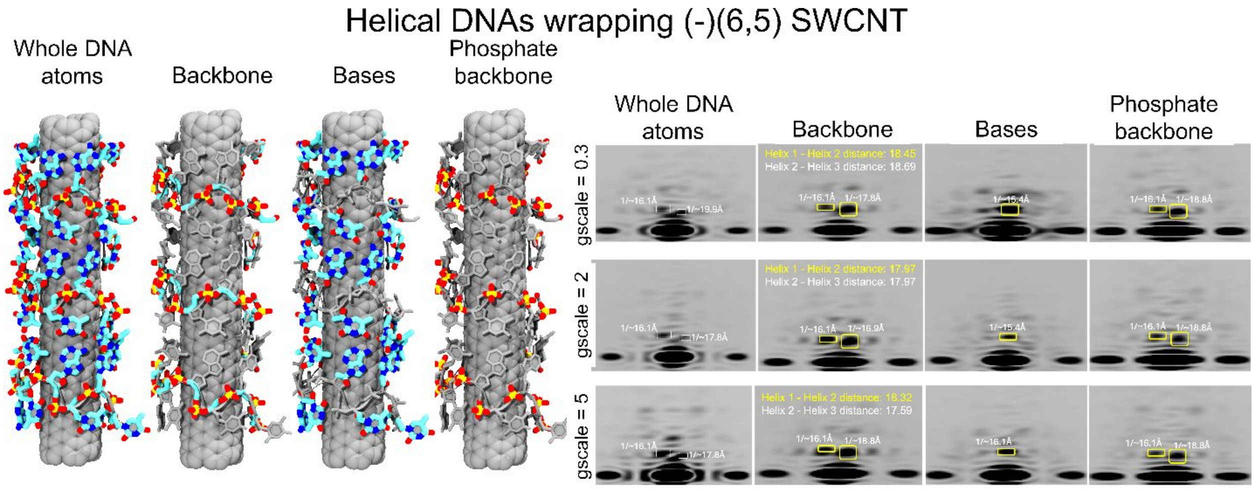

gscale in MDFF simulation	Pitch distances using Whole DNA atoms (Å)	Pitch distances using Backbones (Å) (MD simulation)	Pitch distances using Bases (Å)	Pitch distances using Phosphate backbones (Å)
0.3	- 16.1<br>- 19.9	- 16.1 (18.45)<br>- 17.8 (18.69)	- 15.4<br>-	- 16.1<br>- 18.6
2	- 16.1<br>- 17.8	- 16.1 (17.97)<br>- 16.9 (17.97)	- 15.4<br>-	- 16.1<br>- 18.8
5	- 16.1<br>- 17.8	- 16.1 (18.32)<br>- 18.8 (17.59)	- 16.1<br>-	- 16.1<br>- 18.8

Figure S4. Power spectra of helical DNAs wrapping the (+)(6,5) SWCNT, modeled using DNA structures that include all DNA atoms, only backbone atoms, only base atoms, or only phosphate-backbone atoms. The helical DNA structures were obtained from MDFF simulations performed with gscale values of 0.3, 2, or 5. The top-left panels show representative snapshots of the helical DNA structure around the (-)(6,5) SWCNT from the MDFF simulation with gscale = 2. In each snapshot, the atomic species used for power-spectrum modeling are shown in color, while excluded atoms are rendered in dark tones. The top-right panels display the corresponding modeled power spectra for each atom selection, with the associated gscale value indicated beside each row. The table at the bottom lists the pitch distances extracted from the modeled power spectra shown in the top-right panels. For the pitch distances obtained using backbone-only models, the pitch distances directly computed from the MDFF structures (rather than from the modeled power spectra) based on the center-of-mass (COM) separation between adjacent DNA strands are shown in red. Highlighted entries denote the values reported in the main text.

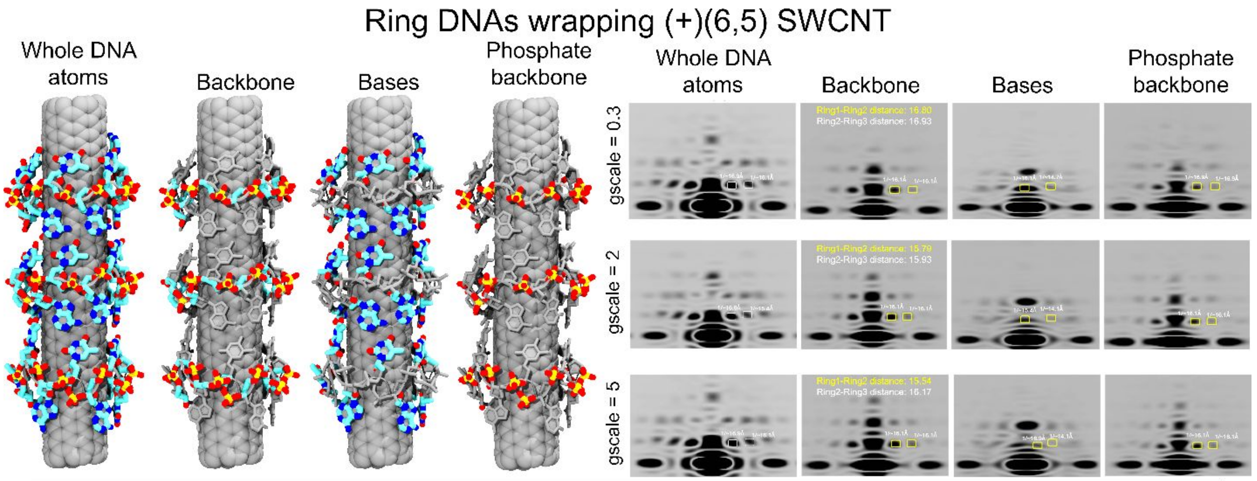

gscale in MDFF simulation	Pitch distances using Whole DNA atoms (Å)	Pitch distances using Backbones (Å) (MD simulation)	Pitch distances using Bases (Å)	Pitch distances using Phosphate backbones (Å)
0.3	- 16.9	- 16.1 (16.8)	- 16.1	- 16.9
	- 16.1	- 16.1 (16.93)	- 14.7	- 16.9
2	- 16.9	- 16.1 (15.79)	- 15.4	- 16.1
	- 15.4	- 16.1 (15.93)	- 14.1	- 16.1
5	- 16.9	- 16.1 (15.54)	- 16.9	- 16.1
	- 16.1	- 16.1 (16.17)	- 14.1	- 16.1

Figure S5. Power spectra of ring DNAs wrapping the (+)(6,5) SWCNT, modeled using DNA structures that include (i) all DNA atoms, (ii) only backbone atoms, (iii) only base atoms, or (iv) only phosphate-backbone atoms. The ring DNA structures were obtained from MDFF simulations performed with gscale values of 0.3, 2, and 5. The top panels and bottom table follow the same formatting and analysis scheme as Figure S4, except that they correspond to the ring DNA-wrapped (+)(6,5) SWCNT system.

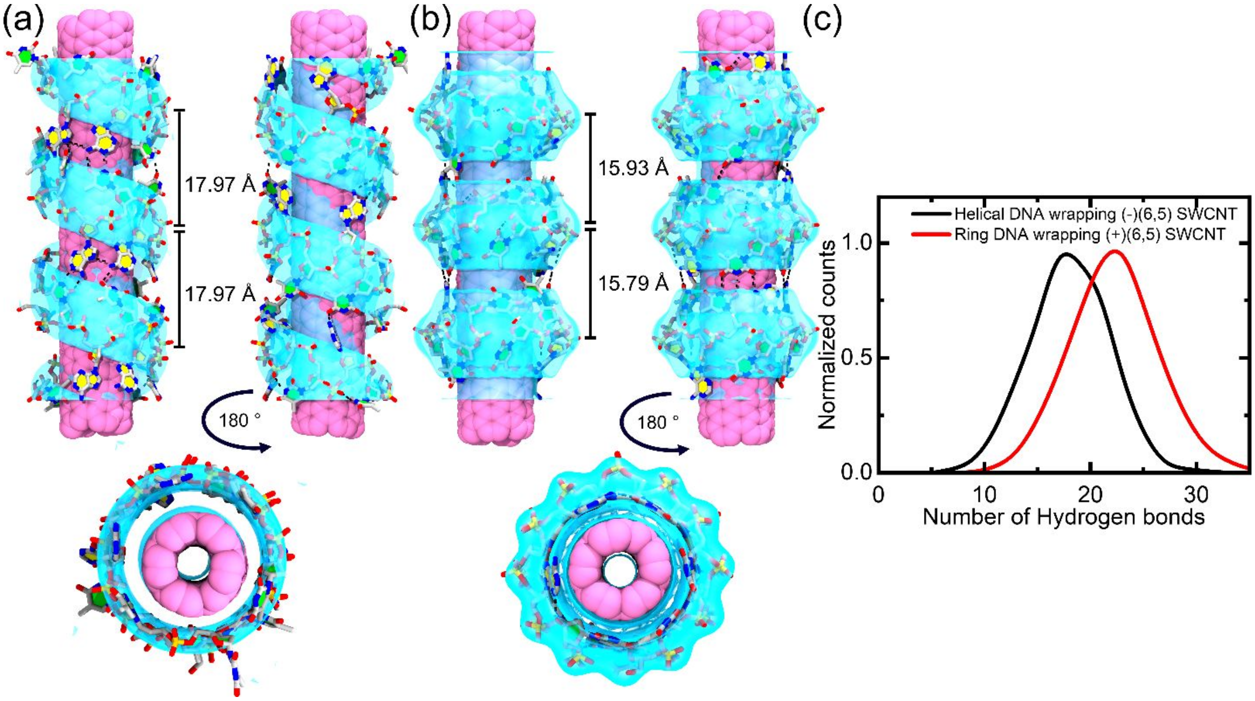

Figure S6. Molecular dynamics flexible fitting (MDFF) simulation snapshots for (a) three helical DNA strands on the (-)(6,5) SWCNT and (b) three ring DNA strands on the (+)(6,5) SWCNT. The top and bottom panels show side and front views, respectively. (c) Normalized distribution curves for the total number of hydrogen bonds, including both inter- and intra-strand interactions, obtained from short 2 ns MD simulations initialized with MDFF-derived structures. Cyan contours represent the experimental cryo-EM density maps. SWCNT carbon atoms are shown in magenta. DNA phosphorus, oxygen, nitrogen, and carbon atoms are depicted in orange, red, blue, and silver, respectively. Black dashed lines indicate hydrogen bonds. The DNA pitch distances computed from MDFF simulations are displayed next to each structure. The experimental pitch distances for the helical and ring DNAs were determined to be 16.4 Å and 15.3 Å, respectively, from the power spectra.

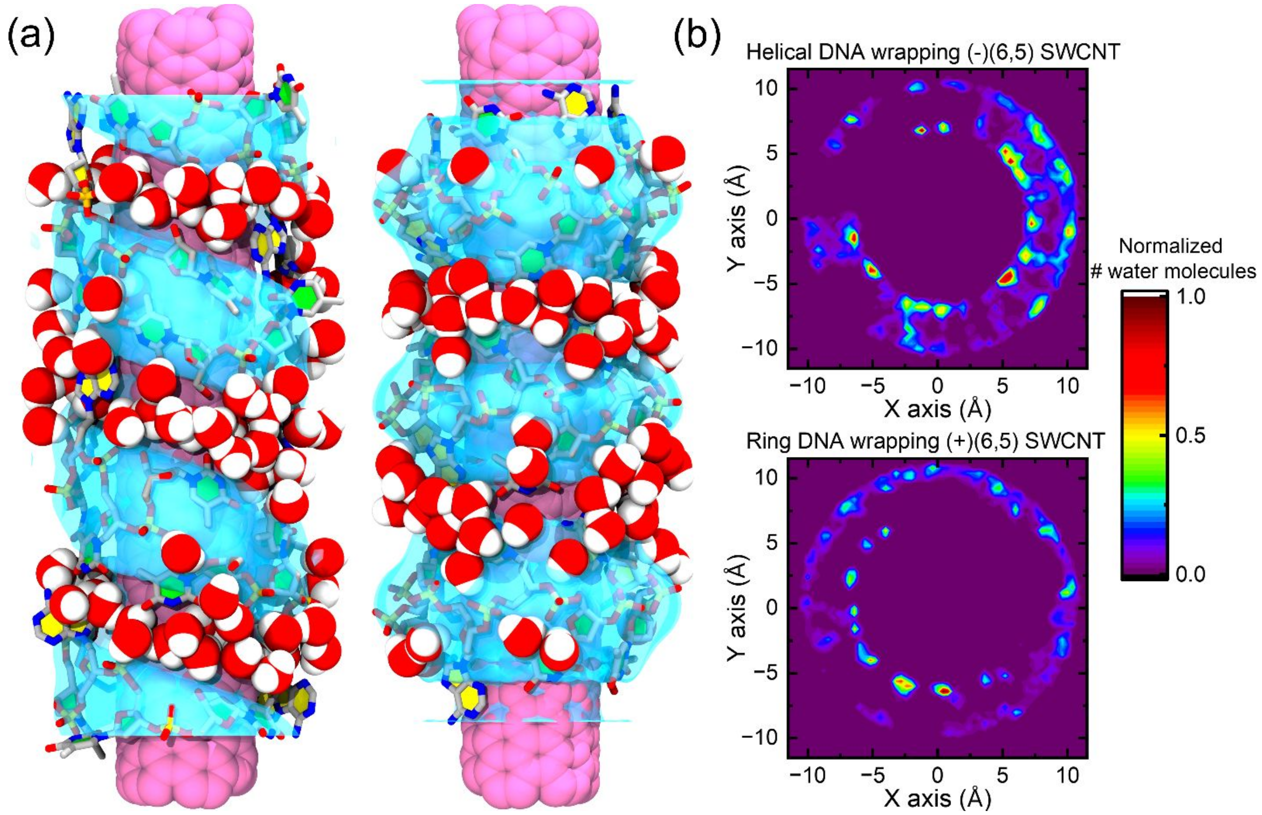

Figure S7. Organization of the first two hydration layers around (6,5) SWCNT enantiomers wrapped by either helical or ring DNA structures. (a) Snapshots from short 2-ns MD simulations initiated from MDFF-derived structures for the (-)(6,5) SWCNT wrapped by helical DNA and the (+)(6,5) SWCNT wrapped by ring DNA. Water molecules within 7 Å of the SWCNT surface are shown. (b) Normalized 2D water-density profiles for the (-)(6,5) and (+)(6,5) SWCNT enantiomers wrapped by helical and ring DNAs, respectively. Color schemes follow those used in Figures S6.

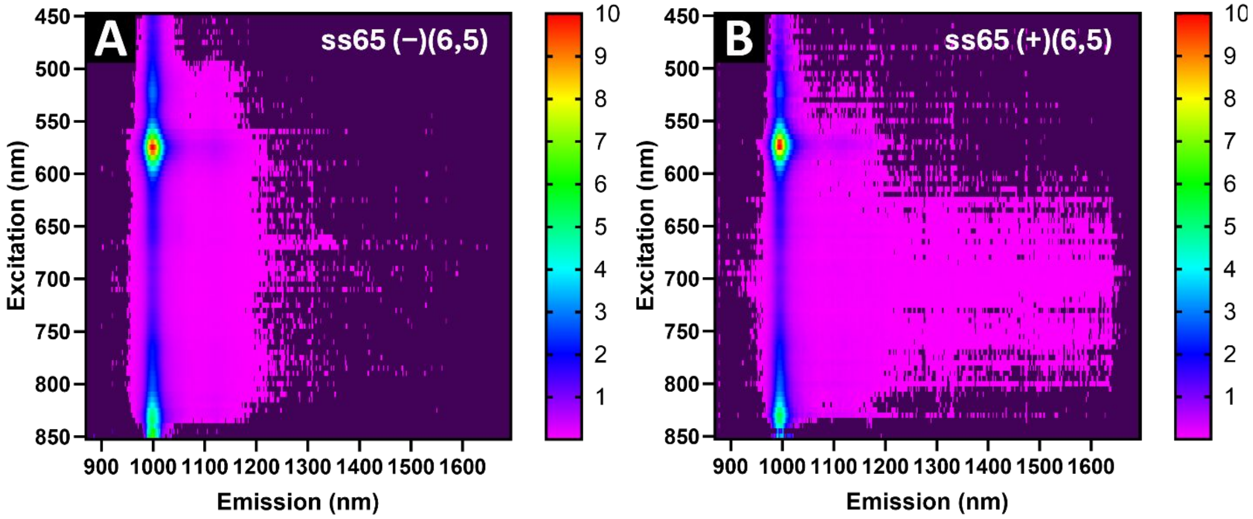

Figure S8. Excitation-emission fluorescence 2D map of (**A**) ss65(-)(6,5), and (**B**) ss65(+)(6,5), respectively.

References

(1) Ao, G.; Khripin, C. Y.; Zheng, M. DNA-Controlled Partition of Carbon Nanotubes in Polymer Aqueous Two-Phase Systems. *J. Am. Chem. Soc.* **2014**, *136* (29), 10383–10392. https://doi.org/10.1021/ja504078b.

(2) Lyu, M.; Meany, B.; Yang, J.; Li, Y.; Zheng, M. Toward Complete Resolution of DNA/Carbon Nanotube Hybrids by Aqueous Two-Phase Systems. *J. Am. Chem. Soc.* **2019**, *141* (51), 20177–20186. https://doi.org/10.1021/jacs.9b09953.

(3) Punjani, A.; Rubinstein, J. L.; Fleet, D. J.; Brubaker, M. A. cryoSPARC: Algorithms for Rapid Unsupervised Cryo-EM Structure Determination. *Nat Methods* **2017**, *14* (3), 290–296. https://doi.org/10.1038/nmeth.4169.

(4) Tang, G.; Peng, L.; Baldwin, P. R.; Mann, D. S.; Jiang, W.; Rees, I.; Ludtke, S. J. EMAN2: An Extensible Image Processing Suite for Electron Microscopy. *Journal of Structural Biology* **2007**, *157* (1), 38–46. https://doi.org/10.1016/j.jsb.2006.05.009.

(5) Humphrey, W.; Dalke, A.; Schulten, K. VMD: Visual Molecular Dynamics. *Journal of Molecular Graphics* **1996**, *14* (1), 33–38. https://doi.org/10.1016/0263-7855(96)00018-5.

(6) Hart, K.; Foloppe, N.; Baker, C. M.; Denning, E. J.; Nilsson, L.; MacKerell, A. D. Jr. Optimization of the CHARMM Additive Force Field for DNA: Improved Treatment of the BI/BII Conformational Equilibrium. *J. Chem. Theory Comput.* **2012**, *8* (1), 348–362. https://doi.org/10.1021/ct200723y.

(7) Denning, E. J.; Priyakumar, U. D.; Nilsson, L.; Mackerell Jr., A. D. Impact of 2′-Hydroxyl Sampling on the Conformational Properties of RNA: Update of the CHARMM All-Atom Additive Force Field for RNA. *Journal of Computational Chemistry* **2011**, *32* (9), 1929–1943. https://doi.org/10.1002/jcc.21777.

(8) Phillips, J. C.; Braun, R.; Wang, W.; Gumbart, J.; Tajkhorshid, E.; Villa, E.; Chipot, C.; Skeel, R. D.; Kalé, L.; Schulten, K. Scalable Molecular Dynamics with NAMD. *Journal of Computational Chemistry* **2005**, *26* (16), 1781–1802. https://doi.org/10.1002/jcc.20289.

(9) Darden, T.; York, D.; Pedersen, L. Particle Mesh Ewald: An N·log(N) Method for Ewald Sums in Large Systems. *J. Chem. Phys.* **1993**, *98* (12), 10089–10092. https://doi.org/10.1063/1.464397.

(10) Beyene, A. G.; Alizadehmojarad, A. A.; Dorlhiac, G.; Goh, N.; Streets, A. M.; Král, P.; Vuković, L.; Landry, M. P. Ultralarge Modulation of Fluorescence by Neuromodulators in Carbon Nanotubes Functionalized with Self-Assembled Oligonucleotide Rings. *Nano Lett.* **2018**, *18* (11), 6995–7003. https://doi.org/10.1021/acs.nanolett.8b02937.

(11) Zheng, Y.; Alizadehmojarad, A. A.; Bachilo, S. M.; Kolomeisky, A. B.; Weisman, R. B. Dye Quenching of Carbon Nanotube Fluorescence Reveals Structure-Selective Coating Coverage. *ACS Nano* **2020**, *14* (9), 12148–12158. https://doi.org/10.1021/acsnano.0c05720.

(12) Alizadehmojarad, A. A.; Zhou, X.; Beyene, A. G.; Chacon, K. E.; Sung, Y.; Pinals, R. L.; Landry, M. P.; Vuković, L. Binding Affinity and Conformational Preferences Influence Kinetic Stability of Short Oligonucleotides on Carbon Nanotubes. *Advanced Materials Interfaces* **2020**, *7* (15), 2000353. https://doi.org/10.1002/admi.202000353.

(13) Alizadehmojarad, A. A.; Bachilo, S. M.; Weisman, R. B. Compositional Analysis of ssDNA-Coated Single-Wall Carbon Nanotubes through UV Absorption Spectroscopy. *Nano Lett.* **2022**, *22* (20), 8203–8209. https://doi.org/10.1021/acs.nanolett.2c02850.

(14) Trabuco, L. G.; Villa, E.; Mitra, K.; Frank, J.; Schulten, K. Flexible Fitting of Atomic Structures into Electron Microscopy Maps Using Molecular Dynamics. *Structure* **2008**, *16* (5), 673–683. https://doi.org/10.1016/j.str.2008.03.005.

(15) Pettersen, E. F.; Goddard, T. D.; Huang, C. C.; Meng, E. C.; Couch, G. S.; Croll, T. I.; Morris, J. H.; Ferrin, T. E. UCSF ChimeraX: Structure Visualization for Researchers, Educators, and Developers. *Protein Science* **2021**, *30* (1), 70–82. https://doi.org/10.1002/pro.3943.
(16) Frank, J.; Shimkin, B.; Dowse, H. Spider—A Modular Software System for Electron Image Processing. *Ultramicroscopy* **1981**, *6* (4), 343–357. https://doi.org/10.1016/S0304-3991(81)80236-7.
